# Physiotherapy and Optimised Enteral Nutrition In the post-acute phase of critical illness (PHOENIX): protocol for a mixed methods feasibility randomised controlled trial

**DOI:** 10.1136/bmjopen-2025-100803

**Published:** 2025-03-27

**Authors:** David McWilliams, Owen Gustafson, Nicola Wyer, Keith Couper, Peter Kimani, Rebecca Kandiyali, Dalia Barghouthy, Rebekah Haylett, Holly Richardson, Miles Negus-Fancey, Elizabeth King, L Gallie, Zudin Puthucheary

**Affiliations:** 1 Centre for Care Excellence, Coventry University, Coventry, UK; 2 Critical Care, University Hospitals Coventry and Warwickshire NHS Trust, Coventry, UK; 3 Oxford Allied Health Professions Research & Innovation Unit, Oxford University Hospitals NHS Foundation Trust, Oxford, UK; 4 University Hospitals Coventry and Warwickshire NHS Trust, Coventry, UK; 5 University of Warwick, Warwick Clinical Trials Unit, Warwick, UK; 6 Critical Care, University Hospitals Birmingham NHS Foundation Trust, Birmingham, UK; 7 Warwick Medical School, University of Warwick, Warwick Clinical Trials Unit, Coventry, UK; 8 Centre for Health Economics (CHEW), University of Warwick, Warwick Clinical Trials Unit, Coventry, UK; 9 Dietetics, University Hospitals Coventry and Warwickshire NHS Trust, Coventry, UK; 10 Patient Representative, England, UK; 11 Critical Care, The Royal London Hospital, London, UK; 12 Williams Harvey Research Unit, Queen Mary University of London, London, UK

**Keywords:** Intensive Care Units, NUTRITION & DIETETICS, Exercise, REHABILITATION MEDICINE, Physical Therapy Modalities

## Abstract

**Introduction:**

Each year in the UK, 140 000 patients are discharged from intensive care units (ICUs) to general hospital wards, almost all with complex rehabilitation needs. 84% of patients still require nutritional support and 98% are not physically independent. Despite this, many are discharged from ICU without a nutrition plan, and failure to recognise malnutrition is common. Consequently, malnutrition persists in the ward environment, leading to poor outcomes and acting as a barrier to successful physical rehabilitation. This transition from intensive care to the ward represents a key stage in the recovery journey, and a window for optimising physical independence prior to hospital discharge, decreasing the need for support in the community. However, uncertainty as to how best to provide ongoing rehabilitation which combines adequate nutrition and exercise on the general ward has driven widespread variation in practice.

We have previously shown the benefits of delivering a structured rehabilitation strategy in the ICU. However, the ward environment poses different challenges to the development of an integrated rehabilitation pathway. There is a need to evaluate the clinical and cost-effectiveness of structured rehabilitation strategies when delivered outside the ICU.

**Methods and analysis:**

Physiotherapy and Optimised Enteral Nutrition In the post-acute phase of critical illness is a bi-centre, mixed methods feasibility randomised controlled trial (RCT). 60 patients will be recruited from ICUs at two acute National Health Service Trusts and randomised on a 1:1 basis to receive either individualised physiotherapy and optimised nutrition post discharge from ICU (intervention) or standard care. The primary objective is to assess the acceptability of the intervention and feasibility of a future, multicentre RCT. The primary outcome measures, which will determine feasibility, are recruitment and retention rates, and intervention fidelity. Acceptability of the intervention will be evaluated through semistructured interviews of participants and staff. Secondary outcome measures include collecting baseline, clinical and outcome data to inform the power calculations of a future definitive trial.

**Ethics and dissemination:**

Ethical approval has been obtained through the Wales Research and Ethics Committee 2 (24/WA/0050). We aim to disseminate the findings through international conferences, international peer-reviewed journals and social media.

**Trial registration number:**

NCT06159868. Prospectively registered on 28 November 2023.

**STRENGTHS AND LIMITATIONS OF THIS STUDY**

- This mixed methods study will evaluate the impact of enhanced physiotherapy and optimised nutrition for survivors of critical illness.
- Qualitative and quantitative approaches will be used to comprehensively assess study outcomes and to inform a main trial and whether or not it would be feasible.
- The outcomes to be assessed by the study are relevant to patients, clinicians and commissioners.
- The recruitment and randomisation of study participants from two sites will increase the generalisability of findings.
- A potential limitation would be the acceptability of the intervention to both patients and staff, which may limit intervention fidelity.

## Introduction

Each year, around 140 000 adults (86% survival rate) are discharged alive from UK intensive care units (ICUs). These individuals frequently have ongoing psychological and physical morbidity, which may persist for many years after discharge.^1^ National guidance developed by the National Institute for Health and Care Excellence and the Intensive Care Society states the need for early and structured rehabilitation for patients admitted to ICU.^2 3^ Despite clear evidence for rehabilitation within the ICU, evidence to support the optimal method for ongoing ward-based rehabilitation is uncertain.

For patients discharged from ICU, suboptimal access to the multidisciplinary team contributes to physical deconditioning and malnutrition. Consequently, on step down to the ward environment, over half of patients experience a deterioration in physical status.^4^ This particularly affects those who leave the ICU most debilitated, with patients who are unable to stand prior to ICU discharge more likely to miss ward-based rehabilitation sessions,^5^ spend longer times in hospital^6^ and are significantly more likely to need ongoing rehabilitation following hospital discharge.^7^ This transition to the ward represents a key stage in the recovery journey, but widespread variation in care across the UK has led to uncertainty as to how best to provide the essential rehabilitation required in the acute setting. High-quality evidence is urgently needed to inform practice and improve outcomes.

The development and widespread implementation of structured, individualised interventions to support patients on ICU step down has the potential to improve overall recovery and quality of life. The James Lind Alliance Intensive Care Priority Setting Partnership highlighted the need for research on supporting recovery after intensive care as a key research priority.^8^

### Rationale for the trial

Patients discharged from intensive care to the ward have complex, multifactorial rehabilitation needs.^9^ To ensure a seamless transition between areas, there is a strong consensus that handover documentation between ICU and wards should be in written form, standardised and structured.^10^,^13^ Despite this, rehabilitation needs are poorly documented in the clinical record, limiting identification and management of ongoing care needs.^10 11 14^ This problem is further compounded due to a lack of cohorting of these patients, who instead are spread throughout the wards in a hospital depending on the original specialty of their underlying admission. This results in widespread variations in care provided, both geographically and in the underlying skills of the receiving wards, meaning post-ICU care commonly misses key patient needs.

Over three-quarters of UK ICUs provide follow-up teams intended to optimise the care of patients in hospital following an ICU stay.^15^ The purpose of follow-up teams is to aid the transition to the ward, support the ward teams with ongoing care such as tracheostomy weaning, improve lines of communication between services and identify clinical deterioration.^2 3^ These teams were often developed with little evidence to support their practice, and the ICU Getting it Right First-Time report and a recent systematic review demonstrated wide variation in provision.^16 17^ The REFLECT study demonstrated follow-up visits failed to ensure key aspects of care were delivered. In addition, follow-up visits usually ceased 24–48 hours after ward transfer despite ongoing clinical problems^18^ limiting the possible impact on post-ICU recovery, and a recent systematic review found no impact on ICU readmission or death.^17^

Two key challenging areas of care provision post-ICU discharge are mobilisation and nutrition. Even in ICUs with established programmes of early mobilisation, around 50% of patients are discharged to the ward unable to stand or transfer to a chair.^19^ Despite these low levels seen at ICU discharge, patient mobility levels actually continue to decrease in the days following step down to the ward.^4 20^ Alongside insufficient handovers of care mentioned above, this is caused by limited provision of rehabilitation in the ward environment, with patients receiving physiotherapy an average of two times per week.^5^ Patients are commonly malnourished at discharge from ICU to the ward.^21 22^ Despite this, many are discharged from ICU without a nutrition plan, and failure to recognise that patients are nutritionally depleted is common.^18^ As a result, inadequate nutrition persists in the ward environment,^23^ with poor outcomes and barriers to rehabilitation directly associated with complex nutritional requirements.^24 25^ The immediate post-ICU discharge phase therefore represents a crucial window, with 98% of patients requiring physiotherapy and 70% being at risk of malnutrition.^9^

In non-ICU populations, increased nutritional support (both protein and energy) has been shown to be cost-effective and associated with improved quality of life and maintenance of functional status.^26^ However, to date, nutrition as an individual intervention, either in the form of protein or energy supplementation, has failed to demonstrate improved outcomes in critically ill patients.^27^ Additionally, exercise alone is catabolic and requires amino acids to stimulate muscle protein synthesis, with energy required for driving such processes and performing exercise.^28^ We hypothesise that a combined approach of structured rehabilitation and optimised nutrition is therefore essential to support recovery in survivors of critical illness.

The Physiotherapy and Optimised Enteral Nutrition In the post-acute phase of critical illness (PHOENIX) mixed methods feasibility randomised controlled trial (RCT) has been designed with the primary aim of assessing the acceptability of the intervention, and recruitment, randomisation and follow-up rates to inform the design of a future adequately powered multicentre trial.

The primary objectives of the feasibility study are

- Proportion of patients agreeing to take part out of all those invited (recruitment rate).
- Proportion of participants who complete the intervention (retention rate).
- Percentage of intervention sessions completed (intervention fidelity).
- Intervention acceptability to participants and service providers.

The secondary objectives are

- To evaluate a range of clinical and patient-reported outcome measures to aid selection of the most appropriate primary outcome measure for a definitive trial, with estimates for sample size calculation and health economic evaluations of any future definitive trial.

## Methods and analysis

### General design

PHOENIX is a two-centre, mixed methods, feasibility RCT with 1:1 randomisation into either intervention or usual care. The trial will be open to recruitment between 1 May 2024 and 31 January 2025. Qualitative interviews will be conducted with participants in the intervention arm and staff involved in delivering the intervention. The trial is being conducted in accordance with the principles of the Declaration of Helsinki and good clinical practice (GCP).

### Participants, interventions and outcomes

#### Study setting

This study is being undertaken in two general adult UK ICUs (University Hospital Coventry and Warwickshire, Coventry and John Radcliffe Hospital, Oxford) which have a proven track record in ICU research.

### Eligibility criteria

Written informed consent is obtained from participants prior to any study procedures taking place (online supplemental file 1). Eligible patients with altered consciousness caused by illness and therapeutic sedation will lack capacity to consent. In this instance, we will approach a personal consultee or an independent registered medical practitioner if no personal consultee is available. Once the participant has recovered from their incapacity, they will be approached to obtain permission to continue in the study. Patients eligible for the study must comply with all of the following before randomisation:

- Adults (≥18 years) who received ≥4 days advanced respiratory support (defined as invasive or non-invasive ventilation) within ICU.
- Alive at ICU discharge.
- Ongoing physiotherapy and dietetic rehabilitation needs identified by the Post-ICU Presentation Screen tool (defined as patients who are unable to transfer from bed to chair independently and not able to meet nutritional requirements independently).

Patients are excluded if they meet any of the following criteria:

- Death expected within the next 72 hours.
- Poor pre-ICU admission mobility (inability to walk>10 m with or without an aid).
- Mobilisation contraindicated (eg, spinal injury).
- Contraindication to or inability to tolerate enteral nutrition.
- Significant acquired brain injury and not recovered to a Glasgow Coma Scale score of ≥14 by ICU discharge.

The inclusion and exclusion criteria are designed to include participants who reflect a general population of patients that have ongoing physical rehabilitation and nutritional needs on discharge from ICU and exclude patients who are unable to mobilise.

### Intervention

The intervention consists of a combination of structured, individualised physiotherapy and optimised nutrition, beginning immediately following recruitment and continuing for up to 14-days or hospital discharge, whichever is sooner. The physiotherapy or nutritional intervention will be terminated early if no longer deemed to be clinically required or appropriate (eg, patent deterioration and receiving end-of-life care; enteral nutrition becomes contraindicated).

The enhanced physiotherapy intervention will be delivered by a dedicated team led by a physiotherapist. The intervention comprises (1) completion of a comprehensive physical and non-physical assessment to create an individualised treatment plan, (2) setting of patient-centred rehabilitation goals with regular reassessment, (3) provision of daily physiotherapy (Monday–Friday), targeting the highest level of mobility attainable and delivery of individualised exercise programmes and (4) close working with ward staff to optimise treatment delivery and ensure seamless transitions of care once the enhanced intervention is complete.

The nutritional component will be led by a specialist dietitian. A comprehensive nutritional assessment will be completed on day one of the intervention period to include current nutritional intake from oral diet, nutritional supplements and enteral tube feeding. Indirect calorimetry (QNRG+) will be used where possible to determine resting energy expenditure, and protein requirements will be calculated using weight-based equations. An individualised nutritional plan will be devised, to include dietary modification, additional food provision, prescription of oral nutritional supplements or modification to enteral feeding regimen as required. This may also include prescribed nutritional supplementation used within 2 hours of the physiotherapy session to address the catabolic nature of exercise. Review and adjustment to the nutritional care plan will occur daily (Monday–Friday) throughout the intervention period. At the end of the intervention period, patients will be transferred to standard dietetic care, with a comprehensive handover provided.

### Usual care

Usual care consists of routine ward-based care including standard dietetic care, mobilisation and rehabilitation interventions, which may include activities of daily living, delivered during normal working hours (between 08:00 and 17:00, Monday–Friday). No further input or involvement will be provided from ICU therapy teams.

### Participant and staff interviews

Semistructured interviews to assess intervention acceptability will be undertaken with a subset of participants in the intervention arm. We will purposively sample participants to ensure a range of age, sex and reason for ICU admission. Participants will be interviewed at a time convenient to them after discharge from hospital. We will also interview staff from the multidisciplinary team that were involved with the delivery of the intervention. Staff may be interviewed at any time during, or shortly after, the intervention period at their site. All interviews will follow a topic guide developed and piloted with input from the patient contributors. The interview content will aim to explore the participants’ and providers’ experiences of receiving and delivering the intervention, barriers to engagement or delivery, and ideas for improving the intervention. It is envisaged that interviews will take up to 45 min.

### Participant timeline

**Figure 1:**
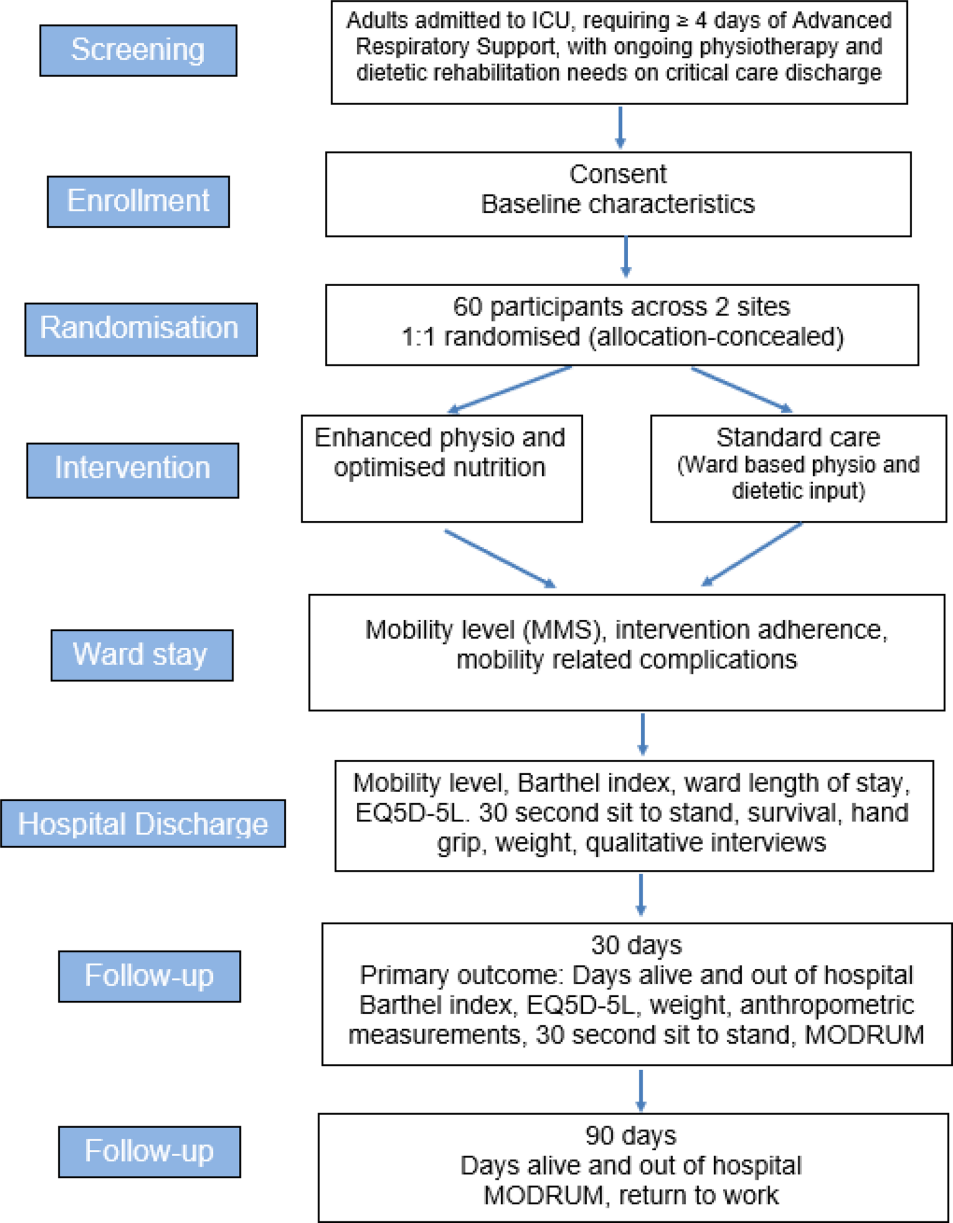
Flow of participants through the study. ICU, intensive care unit; EQ5D-5L, 5 level EQ5D version; MMS, Manchester Mobility Score; MODRUM, Modular Resource Use Measure.

Participant timeline is shown in figure 1.

### Outcome measures

#### Primary outcome measures

- Recruitment rate, overall and by centre.
- Retention rate, defined as the proportion of participants that complete the primary outcome.
- Intervention adherence measured by the proportion of enhanced physiotherapy sessions completed and nutrition delivery compared with targets.
- Acceptability of the intervention to participants and service providers.

#### Secondary outcome measures

Measures that will be used in the future full-scale trial will also be collected at baseline, 14 days or hospital discharge (whichever comes sooner) and at 30 and 90 days following randomisation. The proposed primary outcome for the definitive RCT will be days alive and out of hospital (DAOH) within 30 days. Out of hospital is classified as being at home or usual residence and defined as 30 minus the number of days in hospital (range 0–30), with 0 assigned for death within the 30 days. DAOH is a patient-centred outcome, has been validated with a range of clinical specialties^29^ and takes account of those patients who die or are discharged to community rehabilitation settings.

Further secondary outcomes include:

- Physical function (30 s sit to stand test).
- Functional independence (Barthel Index).
- Quality of Life (EQ-5D-5L).

Additional outcomes collected are:

- DAOH at 90 days.
- Hospital-acquired malnutrition (Global Leadership Initiative on Malnutrition - GLIM criteria).
- Weight and percentage change in weight.
- Time to complete therapy.
- Hospital discharge destination.
- Anthropometric measurements (mid-arm circumference, triceps skin fold, hand grip).

### Feasibility economic evaluation

In the future definitive study, an economic evaluation of the intervention will be conducted from an National Health Service and personal and social services perspective, with results expressed in terms of incremental costs, quality adjusted life years (QALYS) and net monetary benefits and will likely include a lifetime horizon. However, the focus for our economic feasibility study is on optimising the quality of data collected for the first 90 days post randomisation. This is particularly challenging because patients may be stepped down from ICU to different wards, and reporting needs to effectively track the patient’s recovery through a complex system.

The focus for the feasibility study will be on collecting detailed resource use data on the components of this multicomponent intervention (eg, physiotherapist and dietitian staff time; use of rehabilitation aids and dietary interventions) and resources relevant in the broader healthcare system 90 days following randomisation. Details relating to the intervention will primarily be captured by case report forms completed prospectively. Subsequent healthcare resource use (including primary care) related to the patients care (which is not covered by the case report forms CRFs) will be captured via a validated resource use questionnaire (Modular Resource Use Measure), supplemented by some resource use items relevant to the personal social services perspective.

### Sample size

Since this is a feasibility study, the sample size is not determined by a power calculation but rather aims to estimate the rate of recruitment and retention to inform the future trial. We aim to recruit a total of 60 patients, with an equal number (maximum of n=30) from each site and equal random allocation to control and intervention groups. The sample size enables a high probability of continuing to a definitive RCT. For example, for retention rate, progression to definitive RCT will be considered if the estimate is ≥40%. The probabilities of getting a retention estimate ≥40% in each group when the true rates are 50% (low rate) and 80% (a good rate in definitive RCTs) are 0.82 and 1, respectively, which are high. We estimate that we will have sufficient data to identify key issues and themes after having interviewed approximately five members of staff and eight patients from each site unless saturation is deemed to be achieved before this point.

### Assignment of interventions

#### Randomisation

Participants will be randomised in a 1:1 ratio to either the intervention or standard care group using an electronic randomisation system, which will facilitate allocation concealment. Randomisation will be stratified by study site.

#### Blinding

Given the nature of the intervention, it is not possible to fully blind physiotherapists, dietitians or participants to group allocation. However, all assessments will be completed by a team member blinded to randomisation and group allocation. This will reduce the chance of bias throughout the assessment process.

### Data collection, management and analysis

#### Data collection

**Figure 2:**
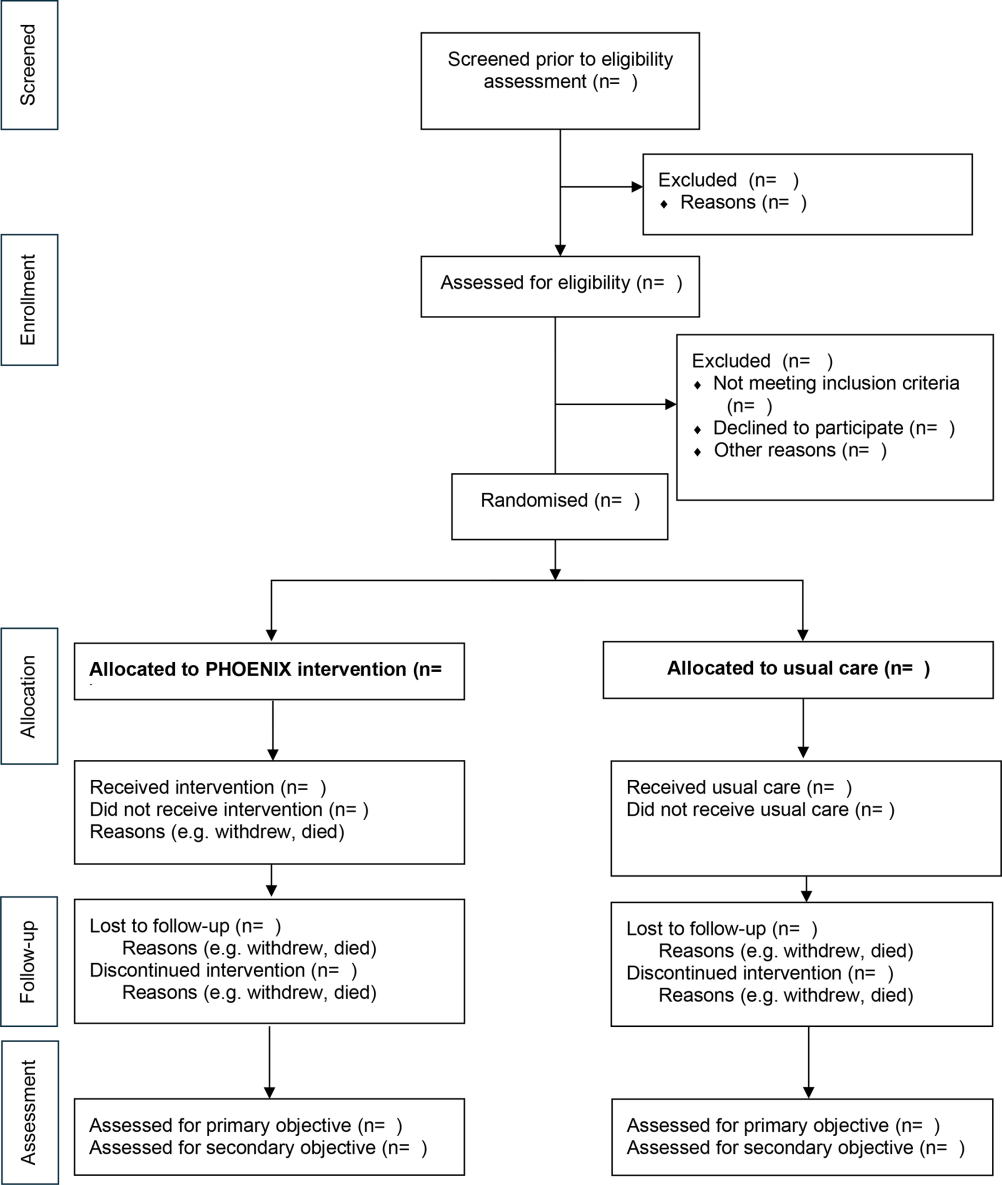
Consolidated Standards of Reporting Trials pilot and feasibility trials flow schematic. PHOENIX, Physiotherapy and Optimised Enteral Nutrition In the post-acute phase of critical illness.

Clinical data are collected at baseline (pre randomisation), daily for up to 14 days post-ICU discharge or hospital discharge (whichever comes sooner) and at 30 and 90 days post randomisation. Data collected are outlined in the study schematic in figure 2 and detailed in table 1.

**Table 1:** Timing of visits and data collection

Baseline (pre randomisation)	Confirmation with inclusion and exclusion criteriaDate and time of consentPatient demographics (age, sex, ethnicity, height, weight)Relevant clinical history (depression, anxiety, comorbidities)Primary admission diagnosisICU length of stayDuration of sedationDuration of mechanical ventilationMMSWeightHospital Malnutrition Score (GLIM criteria)Barthel IndexHand grip strengthMid-arm circumference/triceps skin fold30 s sit to stand
Daily for 14 days or until hospital discharge	Frequency and level of mobilisation (MMS)Nutrition and calories consumedTherapy contacts and durationMobilisation-related complications
End of intervention/hospital discharge	WeightBarthel indexEQ-5D-5LHand grip strength30 s sit to standPost-ICU length of staySemistructured interviewsMortality
30 days post randomisation	WeightEQ-5D-5LGrip strengthMid-arm circumference/triceps skin fold30 s sit to standMortalityResource use (MODRUM)Return to work or normal activity
90 days post randomisation	MortalityResource use (MODRUM)Return to work or normal activity

EQ5D-5L5 level EQ5D versionGLIMGlobal leadership initiative on malnutritionICUintensive care unitMMSManchester Mobility ScoreMODRUMModular Resource Use Measure

In addition to the primary outcome of DAOH, we propose to collect several secondary measures at 30 days and 90 days for evaluation. To enable this, where possible the 30-day and 90-day assessments will be completed face to face in a hospital clinic, if the participants have an appointment scheduled. If this is not possible due to the participant being out of area, unable to travel to the hospital or an appointment is not scheduled, components of the assessment identified will be completed via telephone or video conferencing (outlined in table 1).

### Data management

All data for an individual participant will be extracted from patient charts by the principal investigator or their delegated nominees and recorded in the CRF, either on paper or on the online, validated and GCP compliant electronic data capture (EDC) system. Participant identification in the CRF will be through their unique participant study number allocated at the time of randomisation. Data will be collected from the time the patient is considered for entry into the trial through to their discharge from hospital. If a participant were to pass away or withdraw during the study, identifiable data already collected with consent would be retained and used in the study. A withdrawal form will be completed, and no further data would be collected, or any other research procedures carried out on or in relation to the participant.

Data from the CRF will be entered and stored on the EDC system, as described above. Individual user log-in access to this database will be granted to only those in the study team who require it for the performance of their role. Screening and recruitment logs of all patients approached to take part, and participants enrolled in the trial will be held at each site in secure password-protected files. Paper forms with participant-identifiable information will be held in secure, locked filing cabinets within a restricted area. Audio transcripts from the participant and staff interviews will be recorded on an encrypted device, with transcription completed by a member of the study team.

### Data analysis

Results will be reported in accordance with the Consolidated Standards of Reporting Trials extension to randomised pilot and feasibility trials^30^ and the Consolidated Criteria for Reporting Qualitative Research.^31^

For each of recruitment, retention and outcome measure completion data, rate (as a percentage) and corresponding 95% CI will be computed. Outcomes data (including health economic data) will be analysed descriptively by an appropriate member of the research team using means, range and measures of variance (CIs, SD) for continuous variables, and count (n) and percentages (%) for categorical variables. Distribution of DAOH-30 and DAOH-90 data will be explored to determine the best model to use to analyse them (eg, whether t-test or a model for count data) and to plan a definitive RCT.

Intervention fidelity will be assumed if 70% of planned sessions are completed from those available and broken down by facilitator to examine variation between provider sites. During fidelity scoring, examples of good practice will be identified for training during the main trial. A traffic light system shown in table 2 that is recommended for best practice will be used as a guide for progression to a definitive trial.^32^

**Table 2:** ‘Traffic light’ system to determine progression to a definitive trial

	Green(trial feasible)	Amber(may be feasible with modification)	Red(likely unfeasible)
Recruitment of proposed rate (%)	100%(60 participants)	99%(36–59 participants)	<60%(<36 participants)
Retention rates—defined as collection of primary outcome measure (days alive and out of hospital)	100%(60 participants)	60–99%(36–59 participants)	<50%(<36 participants)
Intervention fidelity (physiotherapy)Proportion of available sessions completed	> 70%	40–70%	<40%
Intervention fidelity (nutrition)Proportion of prescribed supplements taken	>70%	40–70%	< 40%

Verbatim anonymised transcripts of semistructured interviews will be thematically analysed.^33^ Codes and themes will be identified from the data and refined using an iterative process. Analysis of interview transcripts will be supported by NVivo V.12 (NVivo qualitative analysis software; QSR International V.12).

### Patient and public involvement (PPI)

We have planned full involvement across the research cycle. We have collaborated with patient partners to ensure the study addresses key needs that are currently missing from routine care in the recovery of patients following critical illness and that the design is appropriate for potentially anxious and functionally impaired patients. We have identified PPI co-applicants through ICU steps (national patient support group) and the University Hospitals Coventry & Warwickshire PPI group. Both groups expressed strong support for the proposal. Our PPI co-applicants have helped develop the plain English summary, inclusion criteria, personalised intervention and proposed outcomes. Our PPI work has indicated that returning to work (or usual activities) is important in this group. For this reason, our feasibility study will work with our PPI collaborators to develop an acceptable method for capturing this outcome within a future definitive study. We will also work with our PPI group to inform the time points for data collection. They will be full members of the trial team and will assist with analysis and interpretation of the acceptability data (research methods training will be offered), as well as advise on trial delivery and dissemination. All involvement will be reported in the final study report.

## Ethics and dissemination

The study has received a favourable ethical approval from the Wales Research and Ethics Committee 2 (24/WA/0050). Health Research Authority approval was obtained on the 12 March 2024. This paper reports protocol version 2.0 (03 October 2024) and has been written with reference to the Standard Protocol Items: Recommendations for Interventional Trials checklist.^34^

Results from this study will be disseminated at regional and international conferences and in peer-reviewed journals. Authorship of any papers related to this study will follow the ICMJE recommendations (http://www.icmje.org/recommendations/).
