# Supplementary material for: Physiotherapy and Optimised Enteral Nutrition In the post-acute phase of critical illness (PHOENIX): protocol for a mixed methods feasibility randomised controlled trial
Source: BMJ Open. 2025 Mar 27;15(3):e100803. doi: 10.1136/bmjopen-2025-100803 (PMC11956353; doi:10.1136/bmjopen-2025-100803)
Supplement: Supplementary file 1 [file bmjopen-2025-100803.reviewer_comments.pdf]

PEER REVIEW HISTORY

BMJ Open publishes all reviews undertaken for accepted manuscripts. Reviewers are asked to complete a checklist review form and are provided with free text boxes to elaborate on their assessment. These free text comments are reproduced below.

ARTICLE DETAILS

Title (Provisional)

Physiotherapy and Optimised Enteral Nutrition In the post-acute phase of critical illness (PHOENIX): protocol for a mixed-methods feasibility randomised controlled trial

Authors

McWilliams, David; Gustafson, Owen; Wyer, Nicola; Couper, Keith; Kimani, Peter; Kandiyali, Rebecca; Barghouthy, Dalia; Haylett, Rebekah; Richardson, Holly; Negus-Fancey, Miles; King, Elizabeth; Gallie, L; Puthucheary, Zudin

---

This article was not externally reviewed at BMJ Open. Protocols that have been independently assessed prior to submission to BMJ Open are usually fast-tracked to publication on the grounds that further substantial changes will not be possible. This independent assessment will usually be external, independent review for both a substantial grant award from a non-commercial or government funder and ethics approval.
